# Association of Adipocytokines With Carotid Intima Media Thickness and Arterial Stiffness in Obstructive Sleep Apnea Patients

**DOI:** 10.3389/fendo.2020.00177

**Published:** 2020-04-02

**Authors:** Fan Song, Juanjuan Zou, Zhiyuan Song, Huajun Xu, Yinjun Qian, Huaming Zhu, Suru Liu, Jian Guan, Jie Chen, Hongliang Yi

**Affiliations:** ^1^Department of Otolaryngology-Head and Neck Surgery, Shanghai Jiao Tong University Affiliated Sixth People's Hospital, Shanghai, China; ^2^Shanghai Key Laboratory of Sleep Disordered Breathing, Shanghai, China; ^3^Department of Ultrasound in Medicine, Shanghai Institute of Ultrasound in Medicine, Shanghai Jiao Tong University Affiliated Sixth People's Hospital, Shanghai, China

**Keywords:** OSA, chemerin, adiponectin, SFRP5, apelin, IMT, arterial stiffness

## Abstract

**Objective:** Obstructive sleep apnea (OSA) results in increased carotid intima-media thickness (IMT) and arterial stiffness; however, the association between adipocytokines and IMT/arterial stiffness in OSA patients is unclear.

**Methods:** We enrolled 95 normal weight and overweight, not obese, participants from May 2018 to December 2018 in this study. All subjects underwent a carotid artery ultrasound examination and polysomnography. Blood samples were used to determine serum chemerin, adiponectin, SFRP5, and apelin levels. Correlations between two quantitative variables were assessed using the Pearson or Spearman coefficient. Stepwise models of multiple linear regression analysis were performed to assess the independent relationships.

**Result:** IMT in OSA patients was significantly higher than in the non-snorers. There were significant differences in the arterial stiffness parameters such as distensibility coefficient (DC), compliance coefficient (CC), and pulse wave velocity (PWV). SFRP5 level was lower in OSA patients than in non-snorers. Adiponectin correlated with CC, DC, and PWV among OSA patients; however, the relationship disappeared after a multivariable adjustment. Age was independently associated with all quantitative IMT and stiffness indices. AHI and minimum oxygen saturation (Mini SaO_2_) were independently related to arterial stiffness.

**Conclusion:** The quantitative IMT and carotid arterial elasticity were significantly worse among OSA patients. Age was the main independent factor correlated with quantitative IMT and arterial stiffness, and AHI and mini SaO_2_ were associated factors. There were no relationships between aforementioned adipocytokines and quantitative IMT/carotid arterial stiffness.

## Introduction

Obstructive sleep apnea (OSA) is a common sleep-related breathing disorder (1) characterized by recurrent episodes of pharyngeal airway obstruction during sleep (2). The disorder leads to chronic intermittent hypoxia, daytime sleepiness, and sleep fragmentation, which affects over 4% of the general population and 35–45% of obese individuals (3). With regard to OSA and mobility, there exists substantial evidence for a causal relationship between OSA and cognitive impairment-related accidents including an increased risk of traffic accidents. In addition, OSA is related to a higher risk of several cardiovascular diseases such as atrial fibrillation, congestive heart failure, stroke, hypertension, nocturnal arrhythmias, pulmonary hypertension, and atherosclerosis (4–6). Sub-clinical atherosclerotic changes manifesting on arterial walls involve thickening of the intima media and decreased vascular elasticity.

Carotid intima-media thickness (IMT) is an important marker for evaluating early atherosclerosis development (7). Several clinical studies have suggested that an increased IMT is associated with elevated risks of cardiovascular and cerebrovascular diseases (8). It has also been suggested that the values of arterial stiffness and compliance [pulse wave velocity (PWV) and carotid distensibility coefficient (DC)] are useful indices for predicting the future risk of cardiovascular events such as stroke and coronary heart disease (9, 10). Increased IMT in OSA patients has been widely demonstrated (11–14). Innovations in ultrasound technology, such as the US radiofrequency (RF)-data technology, allow for an automatic and accurate measurement of carotid IMT and arterial elasticity. This technology combines the advantages of B-mode imaging (visual morphology) with those of integrated RF-data technology for quantitative assessment of the properties of the blood vessel's walls. It also provides feedback on the quality of the measurement. Several studies have demonstrated that ultrasound radio-frequency (RF) technology can be used as a non-invasive and quantitative method to detect changes in the structure and function of the carotid artery in small vessel disease subjects, in order to evaluate preclinical atherosclerosis (15, 16). In addition, this technology has been applied in many previous clinical studies (17, 18). However, it is rarely used for OSA patients.

Moreover, changes in the levels of adipocytokines such as chemerin, apelin, and secreted frizzled-related protein5 (SFRP5) are important risk factors for atherosclerosis and cardiovascular diseases (19, 20). The relationship between OSA and these factors however remain unclear. Previous studies on adiponectin and apelin levels in OSA patients report conflicting results (21–24) while other few focusing on chemerin and SFRP5 in OSA patients, have been limited by the absence of multivariate adjustment or a smaller sample size (25, 26). Furthermore, the relationship between these adipokines and IMT among OSA patients was not clarified.

Therefore, we conducted this study among OSA patients with BMI <30 kg/m^2^ to (1) determine the correlation between OSA and chemerin, apelin, adiponectin, and SFRP5; (2) examine the relationship between adipokines and IMT/arterial stiffness.

## Methods

### Participants

OSA patients were consecutively recruited at the Department of Otolaryngology, Head and Neck Surgery of Shanghai Sixth People's Hospital from May 2018 to December 2018. The inclusion criteria were: (1) aged between 20 and 55 years old and, (2) BMI between 18 and 30 kg/m^2^. The exclusion criteria were as follows: (1) coronary atherosclerotic cardiopathy, cerebrovascular disease, and other peripheral vascular diseases; (2) post-percutaneous transluminal coronary angioplasty or bypass surgery; (3) renal failure requiring hemodialysis or peritoneal dialysis; (4) chronic obstructive pulmonary disease, chronic cardiac failure, and diabetes mellitus; (5) hepatic encephalopathy and liver carcinoma and, (6) missing data. In total, 62 OSA patients were included in the study. In addition, we recruited 33 healthy volunteers without snoring from the general population. Written informed consent was obtained from all subjects following the guidelines of the National Ethics Regulation Committee and was in accordance with the Declaration of Helsinki. This study was approved by the Internal Review Board of the Institutional Ethics Committee of the Shanghai Jiao Tong University Affiliated Sixth Hospital [2018-KY-021(K)].

### Polysomnography

To acquire objective information on sleep parameters, polysomnography (Alice 4 or 5, Respironics Inc.; Pittsburgh, PA, USA) was performed overnight between 22:00 and 06:00 and included electrooculography, electroencephalography (EEG), electromyography (EMG), and electrocardiography. Airflow was measured using nasal and oral thermistors. We also monitored the thoracic-abdominal movements, performed finger pulse oximetry, assessed snoring using a tracheal microphone, and recorded changes in body position. Apnea was defined as a complete cessation of airflow for at least 10s (27) when measured using a nasal cannula and thermistors. Hypopnea was defined as a ≥50% reduction in the airflow accompanied by a decrease in oxyhemoglobin saturation of ≥3%, or arousal indicated by the electroencephalogram. The average number of apnea and hypopnea episodes per hour of sleep time was calculated as the apnea-hypopnea index (AHI). The percentage of total sleep time spent with SaO_2_ <90% and minimal oxygen saturation (Mini SaO_2_) were also recorded. Based on the American Academy of Sleep Medicine criteria (28), AHI ≥5 was defined as OSA.

### Anthropometric Measurements

Weight was measured using a weighing scale with the subject standing still in lightweight clothing and bare feet. Height was measured as the maximum distance from the feet to the highest point on the head while standing without any footwear and feet kept together. The body mass index (BMI) was calculated as weight (kilograms)/height squared (square meters).

### Common Carotid Artery Measurements

The MyLab Twice (Esaote, Genoa, Italy) ultrasound machine used was equipped with a 12-MHz vascular probe (LA523), which measured quantitative IMT, and quantitative arterial stiffness (QAS) capabilities automatically. The software used a complex algorithm that could process all data from the lesion as RF signals. It is suitable for the quantitative evaluation of the vessel wall's properties. All participants underwent an examination of the IMT and arterial stiffness of the bilateral common carotid artery (CCA) in the supine position with a head elevation ≤ 45° and a side tilt of 30° in order to fully expose the neck. The internal diameter of the CCA was measured in a longitudinal section. All the measurements were performed by one investigator (JLX), who was blinded to the OSA diagnosis.

**Figure 1:**
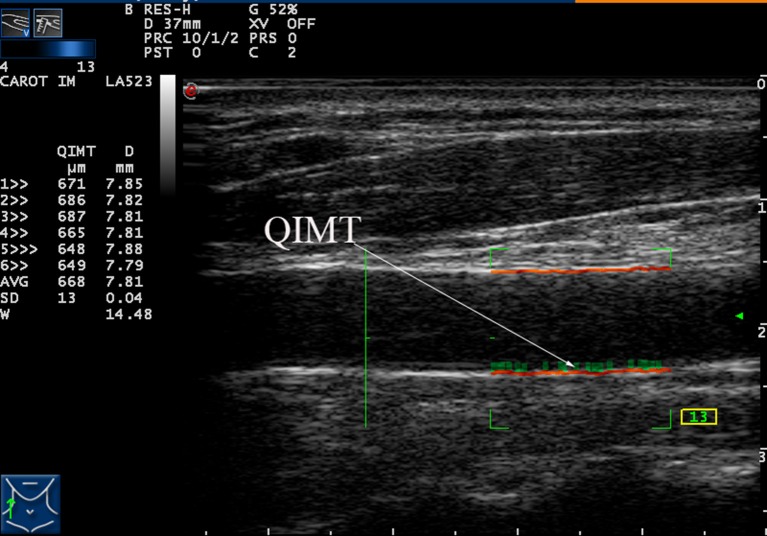
Measurement of the intima-media thickness (IMT) of the right carotid artery by radio frequency data technique.

Each patient underwent automatic bilateral common carotid arterial measurements over six cardiac cycles. Once all six met the quality standard (quality control shown as a green number on-screen during scanning), their average was calculated and used as a final result for that patient. These measurements and average calculations were performed automatically and displayed on the left side of the screen, as shown in Figure 1.

Quantitative IMT measurements were performed in a longitudinal section, strictly perpendicular to the ultrasound beam, with both arterial walls clearly visualized. A high-quality standard B-mode image was acquired and the distal 1–1.5 cm of the bilateral CCA just proximal to the bulb was measured using a computer analysis system. Automatic quantitative IMT calculation was measured in real-time by the radiofrequency reception signal (Figure 1) and the average of the three measurements was taken as the final result.

**Figure 2:**
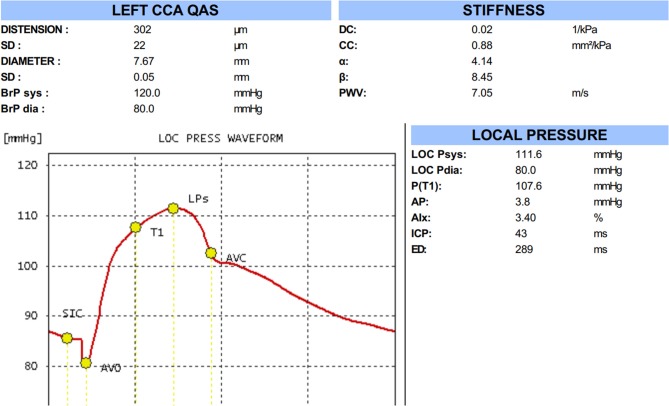
The result of quality arterial stiffness (QAS) test of the left carotid artery by radio frequency data technique.

Automatic QAS measurements were performed on the same common carotid arterial segments as those used for the quantitative IMT measurement, evaluating the modification of the arterial internal diameter between the systolic and diastolic phases as follows: Carotid arterial internal diameter waveforms were assessed using an ultrasound and converted to carotid arterial pressure waveforms using a derived exponential relationship between the pressure and arterial cross-sections; derived carotid arterial pressure waveform was calibrated to the brachial end-diastolic pressure and MAP by iteratively changing the wall rigidity coefficient (Figure 2). QAS was measured automatically over six cardiac cycles. QAS data analysis software also calculated the following carotid arterial stiffness indices: local systolic and diastolic blood pressures, pulse wave velocity (PWV), compliance coefficient (CC), distensibility coefficient (DC), and stiffness index (α*&β*) according to the following formulae: $PWV=\frac{1}{\sqrt{\rho^{*}DC}}$; CC = π (Ds^*^Ds-Dd^*^Dd)/[4(Ps-Pd)]; α = ln(Ps/Pd)/[(As-Ad)/Ad]; β = ln (Ps/Pd)/[(Ds-Dd)/Dd], where As = systolic area, Ad = diastolic area, Ds = systolic diameter, Dd = diastolic diameter, Ps = systolic blood pressure, Pd = diastolic blood pressure, and P = blood density.

### Blood Sampling and Laboratory Analysis

Fasting venous blood samples were collected in the morning after polysomnography from the antecubital vein, after at least 8 h of overnight fasting, and immediately centrifuged (5 min at 3,000 rpm). Fasting triglycerides (TG), total cholesterol (TC), high- and low-density lipoprotein (HDL and LDL, respectively) were measured using a random-access chemistry analyzer (Hitachi 7600, Japan). The remaining serum samples were cryopreserved at −80°C until further analysis. Chemerin and adiponectin serum concentrations were measured using commercially available ELISA kits (Abcam, Bristol, UK, ab155430 and ab108786, respectively) following the manufacturer's instructions. Apelin (Cusabio, China, CSB-E14334 h) and SFRP5 (USCN Life Science, Wuhan, P.R.China) were also detected by ELISA kits. Intra-assay and inter-assay CVs were <10 and <12% for chemerin, 3 and 8.3% for adiponectin, <8 and <10% for apelin, and <10 and <12% for SFRP, respectively.

### Statistical Analyses

All statistical analyses were performed using the SPSS software version 20.0 for Windows (IBM Corp., Armonk, NY, USA). For the CCA parameters, we calculated the average of bilateral CCA before analysis. The distributions of quantitative variables were tested for normality using the Kolmogorov–Smirnov test and the QQ plot was used for vision tests. Normal data are presented as mean ± standard deviation (SD) and skewed data are described as median [interquartile range (IQR)]. The categorical variables are expressed as a number (percentage). The variables were compared between the non-snoring group and OSA group using an independent *t*-test for normalized data and Mann-Whitney *U*-test for non-normalized data. All categorical variables were analyzed with Chi-square or Fisher's exact test, when appropriate. Correlation between two quantitative variables was assessed using the Pearson or Spearman coefficient. Stepwise models of multiple linear regression analyses were performed in order to assess the impact of independent variables on mean CCA parameters. Results were considered statistically significant if the *p*-value was <0.05.

## Results

### Basic Characteristics of the Study Population

The basic characteristics of the 95 subjects, consisting of 62 OSA patients and 33 non-snoring participants, were collected. As shown in Table 1, there were no significant differences between the two groups in terms of age, BMI, sex, HDL, chemerin, adiponectin, apelin, or stiffness coefficients α and β. The serum levels of TG, TC, and LDL and the sleep parameters AHI and CT90 were significantly higher in OSA patients than the non-snorers. Moreover, the mini SaO_2_ was much more severe among OSA patients. The serum SFRP5 levels were significantly lower in OSA patients than the non-snoring participants. As for the CCA parameters, the quantitative IMT, stiffness coefficients (α and β) and PWV were significantly higher in the OSA patients than the non-snoring participants. CC and DC in OSA patients were significantly lower than those in non-snoring participants. There was no significant difference in the stiffness coefficients (α and β) between the two groups.

**Table 1:** Baseline characteristics and laboratory data of participants.

	**Non-snorers (*N* = 33)**	**OSA patients (*N* = 62)**	***p*-value**
Age (years)	37.51 ± 10.87	40.38 ± 9.50	0.185
BMI (kg/m^2^)	23.30 ± 3.07	24.18 ± 1.55	0.132
Males (*n*, %)	28(84.8)	58 (93.5)	0.168
TG (mmol/L)	1.07 ± 0.53	1.88 ± 1.05	<0.001
TC (mmol/L)	4.07 ± 0.91	4.63 ± 0.76	0.002
LDL (mmol/L)	2.24 ± 0.67	2.65 ± 0.67	0.005
HDL (mmol/L)	1.05 ± 0.29	1.02 ± 0.28	0.568
AHI (events/h)	1.78 ± 1.41	29.11 ± 20.45	<0.001
Mini SaO_2_ (%)	90.74 ± 5.33	79.31 ± 8.78	<0.001
CT90 (%)	0	1.5(0.1,4.8)	<0.001
Chemerin (ng/mL)	99.43 ± 21.03	109.27 ± 25.58	0.064
Adiponectin (μg/mL)	41.80 ± 21.63	38.92 ± 18.51	0.525
SFRP5 (ng/mL)	3.25 ± 1.36	2.38 ± 1.01	0.001
Apelin (ng/mL)	0.88 ± 0.64	0.75 ± 0.52	0.280
Quantitative IMT (mm)	0.49 ± 0.09	0.53 ± 0.10	0.041
CC (mm^2^/kPa)	1.17 ± 0.52	0.96 ± 0.36	0.026
DC (1/KPa)	0.03 ± 0.01	0.02 ± 0.01	0.011
α	3.5 ± 1.71	4.17 ± 1.89	0.12
β	7.26 ± 3.43	8.51 ± 3.81	0.12
PWV (m/s)	6.15 ± 1.41	6.91 ± 1.49	0.019

*BMI, body mass index; TG, triglyceride; TC, total cholesterol; HDL, high-density lipoprotein, LDL, low-density lipoprotein; AHI, apnea-hypopnea index; Mini SaO_2_, minimum arterial oxygen saturation; CT90, percentage of sleep duration with SpO_2_ <90%, SFRP5, secreted frizzled-related protein 5; QIMT, quality intima-media thickness; CC, compliance coefficient; DC, distensibility coefficient; PWV, pulse wave velocity*.

### Correlation for Quantitative IMT, Arterial Elasticity, Adipokines, and Other Variables

The correlations between quantitative IMT, arterial stiffness, and participants' characteristics are listed in Table 2. Age, TG, TC, AHI, and CT90 were significantly correlated to quantitative IMT. CC was negatively correlated with age, the proportion of males, AHI, and CT90, but positively correlated with mini SaO_2_ and adiponectin. As for DC, it was negatively correlated with age, the proportion of males, TG, AHI, and CT90% and positively correlated with mini SaO_2_ and adiponectin. The arterial stiffness coefficients, α and β, were both positively correlated with age, AHI, and CT90 and negatively correlated with mini SaO_2_. PWV was positively correlated with age, AHI, and CT90 and negatively related to mini SaO_2_ and levels of adiponectin.

**Table 2:** Correlation between CCA parameters and clinical, biochemical, and sleep characteristics.

	**Quantitative IMT**		**CC**		**DC**		**α**		**β**		**PWV**	
	***r***	***p***	***r***	***p***	***r***	***p***	***r***	***p***	***r***	***P***	***r***	***p***
Age	0.540	<0.001	−0.545	<0.001	−0.617	<0.001	0.605	<0.001	0.602	<0.001	0.653	<0.001
BMI	0.05	0.632	−0.123	0.233	−0.011	0.915	0.063	0.545	0.061	0.559	0.085	0.413
Males	0.089	0.393	−0.228	0.026	−0.337	0.001	0.179	0.082	0.179	0.083	0.255	0.012
TG	0.243	0.02	−0.162	0.125	−0.218	0.038	0.105	0.323	0.103	0.323	0.193	0.067
TC	0.246	0.017	−0.081	0.054	−0.105	0.313	0.013	0.9	0.012	0.911	0.085	0.416
LDL	0.201	0.052	−0.008	0.941	−0.39	0.707	−0.005	0.960	−0.006	0.952	0.014	0.891
HDL	−0.079	0.448	0.098	0.346	0.138	0.186	−0.105	0.316	−0.104	0.320	−0.161	0.122
AHI	0.239	0.019	−0.273	0.007	−0.295	0.004	0.204	0.048	0.204	0.048	0.299	0.003
Mini SaO_2_	−0.188	0.068	0.351	0.028	0.348	0.001	−0.321	0.001	−0.321	0.001	−0.410	0.001
CT90	0.296	0.004	−0.351	<0.001	−0.390	<0.001	0.358	<0.001	0.360	<0.001	0.404	<0.001
Chemerin	0.014	0.899	0.128	0.229	−0.044	0.678	−0.029	0.783	−0.030	0.780	−0.017	0.876
Adiponectin	−0.097	0.379	0.277	0.01	0.311	0.004	−0.157	0.151	−0.156	0.155	−0.236	0.03
SFRP5	0.05	0.637	−0.131	0.218	0.138	0.195	0.060	0.575	0.058	0.575	0.094	0.379
Apelin	−0.077	0.478	0.1	0.354	0.168	0.118	0.007	0.947	0.008	0.942	−0.033	0.760

*Spearman coefficient for Arousal Index and CT90; Pearson coefficient for other variables*.

*CCA, common carotid artery; BMI, body mass index; TG, triglyceride; TC, total cholesterol; HDL, high-density lipoprotein, LDL, low-density lipoprotein; AHI, apnea-hypopnea index; Mini SaO_2_, minimum arterial oxygen saturation; CT90, percentage of sleep duration with SpO_2_ <90%, SFRP5, secreted frizzled-related protein 5; QIMT, quality intima-media thickness; CC, compliance coefficient; DC, distensibility coefficient; PWV, pulse wave velocity*.

As shown in Table 3, chemerin was positively correlated with BMI. A negative correlation between adiponectin and age, the proportion of males, and TG was observed. Furthermore, serum SFRP5 levels were negatively correlated with LDL and AHI. Serum apelin levels were only significantly correlated with CT90.

**Table 3:** Correlation between adipokines and clinical, biochemical, and sleep characteristics.

	**Chemerin**		**Adiponectin**		**SFRP5**		**Apelin**	
	***r***	***p***	***r***	***p***	***r***	***p***	***R***	***P***
Age	−0.032	0.763	−0.255	0.018	0.203	0.055	−0.132	0.221
BMI	0.292	0.005	−0.066	0.549	−0.019	0.859	0.07	0.515
Males	0.045	0.677	−0.382	<0.001	0.182	0.087	0.138	0.200
TG	0.077	0.483	−0.355	0.001	0.201	0.063	−0.017	0.881
TC	−0.055	0.608	−0.190	0.083	−0.022	0.840	−0.096	0.379
LDL	0.116	0.279	−0.028	0.530	−0.229	0.031	−0.125	0.250
HDL	−0.075	0.485	0.091	0.411	−0.164	0.125	0.085	0.434
AHI	0.206	0.052	−0.173	0.113	−0.209	0.048	−0.092	0.396
Mini SaO_2_	−0.196	0.064	0.186	0.088	0.133	0.210	0.073	0.497
CT90	0.207	0.051	−0.136	0.214	−0.184	0.083	−0.257	0.012

*Spearman coefficient for CT90; Pearson coefficient for other variables*.

*BMI, body mass index; TG, triglyceride; TC, total cholesterol; HDL, high-density lipoprotein, LDL, low-density lipoprotein; AHI, apnea-hypopnea index; Mini SaO_2_, minimum arterial oxygen saturation; CT90, percentage of sleep duration with SpO_2_ <90 %, SFRP5, secreted frizzled-related protein 5*.

### Independent Relationship on Quantitative IMT and Arterial Stiffness

Based on the correlation analysis, we employed multiple linear regression analyses to identify variables that were critical determinants of quantitative IMT and arterial stiffness. Results from the stepwise analysis revealed that age was independently associated with all of the quantitative IMT and stiffness indices. In addition to this, there was a significant association between TC and quantitative IMT, between mini SaO_2_ and CC, and between DC and sex/AHI; mini SaO_2_ was independently associated with arterial stiffness coefficients, α and β; and sex and mini SaO_2_ were independently associated with PWV (Table 4).

**Table 4:** Multivariate linear regression analyses for quantitative IMT and stiffness.

	**Quantitative IMT**		**CC**		**DC**		**α**		**β**		**PWV**	
	**B**	***P***	**B**	***P***	**B**	***P***	**B**	***P***	**B**	***P***	**B**	***P***
Age	0.511	<0.001	−0.465	<0.001	−0.536	<0.001	0.560	<0.001	0.557	<0.001	0.560	<0.001
Males	–	–	–	–	−0.273	0.001	–	–	–	–	0.182	0.024
AHI	–	–	–	–	−0.17	0.048	–	–	–	–	–	–
Mini SaO_2_	–	–	0.237	0.013	–	–	−0.186	0.028	−0.186	0.028	−0.253	0.003
TC	0.213	0.017	–	–	–	–	–	–	–	–	–	–

*AHI, apnea-hypopnea index; Mini SaO_2_, minimum arterial oxygen saturation; TC, total cholesterol*.

## Discussion

The present study demonstrates that OSA patients have a significantly increased quantitative IMT, decreased carotid arterial elasticity, and a significantly decreased SFRP5. Adiponectin correlated with CC, DC, and PWV among OSA patients but this relationship disappeared after the multivariable adjustment. Furthermore, age is the main independent factor correlated with quantitative IMT and arterial stiffness. AHI and mini SaO_2_ were also independently associated with arterial stiffness.

The structural and functional alterations in arteries have been of interest for several years as they are considered to be risk factors for cardiovascular diseases, such as atherosclerosis (19, 20). As OSA is one of the most common causes of cardiovascular diseases, we concentrated on the CCA changes caused by OSA and evaluated the arterial lesions using a relatively new method known as the ultrasound RF data technology. Notably, it was reported that about 10% of hospitalized stroke patients were aged 55 years or younger (29), so we excluded patients over 55 years of age in consideration the purpose of our research was to explore the relationships between IMT, arterial stiffness and adipokines among younger and middle-aged population. Previous research indicates that quantitative IMT and QAS techniques, as non-invasive examinations, provides a comprehensive evaluation of CCA remodeling for assessing preclinical atherosclerosis and early-stage diabetes (15, 16, 30). Although several studies have shown that there are no changes in carotid IMT in OSA patients compared to non-snorers (13, 31), substantial evidence indicates a significant increase in OSA patients compared to non-snorers (32). In this study, we creatively used the ultrasound radio-frequency technology to detect changes in the structure and function of the carotid artery in OSA patients. We observed that the carotid quantitative IMT was significantly increased, while the arterial elasticity was significantly decreased in OSA patients compared to the non-snorers. It is widely acknowledged that OSA can directly worsen endothelial functions. In this study, AHI was independently correlated with DC and mini SaO_2_ was independently associated with CC, stiffness coefficients, α and β, and PWV. The two primary mechanisms underlying endothelial abnormalities in OSA are the repetitive episodes of hypoxia/reoxygenation associated with a transient cessation of breath during sleep and the sleep fragmentation/deprivation (33). These two mechanisms not only lead to the onset of systemic inflammation but also result in oxidative stress. Chang et al. reported that inflammation and oxidative stress play an important role in the increasing carotid IMT in OSA patients (11).

Adiponectin is an adipokine secreted by the adipocytes, which is closely related to metabolism and may have both, antiatherogenic and anti-inflammatory properties. Previous studies on adiponectin levels in OSA patients report conflicting results. Wolk et al. reported that adiponectin levels were higher in OSA patients compared to non-snorers (21), while other researchers reported the opposite (22). It has been suggested that adiponectin is associated with subclinical atherosclerosis (34–36). Lower adiponectin levels were independently associated with elevated IMT, a marker of early-stage atherosclerosis, suggesting that adiponectin may protect against atherosclerosis. We observed that adiponectin was positively associated with CC and DC but negatively associated with PWV. In this study, the serum adiponectin levels in OSA patients were not different from those of the non-snorers. We assume that OSA patients may have lost the protective effect of adiponectin, which may be reduced due to inflammation and oxidative stress however this requires further investigation for confirmation.

Apelin, a peptide isolated from bovine stomach extract in 1998, is a classical endogenous ligand for APJ (37). Apelin is secreted from the white adipose tissue and plays an important role in energy metabolism and insulin resistance. Moreover, it may have a critical effect in respiratory physiology but only a few studies have investigated the levels in OSA patients and report conflicting conclusions (23, 24). Sabry et al. (38) reported that serum apelin, which is positively correlated with carotid IMT, can be a new marker of early atherosclerosis in children with Type 1 diabetes mellitus. Our study is the first to compare apelin levels between OSA patients and non-snorers, focusing on the relationship between apelin levels, quantitative IMT, and arterial elasticity in OSA patients. However, there was no significant difference in terms of apelin levels between the OSA and non-snoring groups, and no correlation between apelin, quantitative IMT, and arterial stiffness.

Chemerin is a newly identified adipokine and an emerging critical regulator of several biological processes, including adipogenesis, energy metabolism, and immune responses (39, 40). To date, only one study has examined the relationship between OSA and chemerin (41). In this before mentioned study, which focused only on an extremely obese population, the serum levels of chemerin in OSA patients were not different from those of non-snorers. In this study, focusing on non-obese population, the serum chemerin levels were similar in the OSA patients and non-snorers. BMI was the only variable that correlated with chemerin and there was no significant difference in the BMI between the two groups. Recently, several studies have reported that elevated circulating chemerin levels correlate with endothelial dysfunction and can be a biomarker of subclinical atherosclerosis (42–44). However, in our study, no such relationship between the chemerin levels, quantitative IMT, and arterial stiffness was observed.

SFRP5, a novel adipokine secreted by the adipocytes has anti-inflammatory effects and reduces insulin resistance (45). Only one study has examined the relationship between SFRP5 and OSA and reported no differences in the SFRP5 levels between the OSA patients and non-snorers (46). In our study, the serum SFRP5 levels in OSA patients were significantly lower than those of the non-snoring group. Besides, SFRP5 correlated with LDL and AHI. As a previous study reported, SFRP5 levels are negatively associated with IMT and arterial stiffness reflected by PWV (47). In Miyoshi's research, lower serum SFRP5 levels were significantly correlated with an increased risk of coronary artery disease (48). SFRP5 exerts anti-inflammatory effects through the suppression of the non-canonical WNT5/JNK signaling pathway (49, 50), which can stimulate endothelial cell proliferation (51) and vascular calcification (52) associated with the pathogenesis of atherosclerosis. Our research suggests that SFRP5 is affected by AHI. SFRP5 levels of OSA patients were significantly lower than those of the non-snoring group, suggesting that lower SFRP5 may contribute to the risk of coronary artery disease in OSA patients.

There are many other adipokines, such as leptin, resistin, etc. Leptin, a crucial factor in regulating metabolism and body weight, is considered to be a proinflammatory adipokine (53). In a recent study, Bingol et al. demonstrated that the leptin level was higher in patients with severe obesity and that severe OSA patients had a lower leptin level (54). However, Berger et al. reviewed many of these studies and determined that OSA can augment leptin release from adipose tissue, which results in hyperleptinemia (55). Furthermore, hyperleptinemia and leptin resistance promotes the generation of reactive oxygen species, increasing oxidative stress, and promoting inflammation, which increases cardiovascular risk (55). However, some studies found no significant difference in the serum leptin levels between non-obese moderate/severe OSA patients and non-obese simple snoring/mild OSA patients. The CIMT is positively correlated with serum leptin levels in patients with psoriasis (56) and premenopausal systemic lupus erythematosus patients (57). Lambert et al. indicated that an obvious C-IMT regression is associated with a reduction in leptin levels, independent of the weight loss (58). However, the correlation between leptin and IMT in OSA patients is still unclear and requires further investigation.

Resistin is an adipokine with pro-inflammatory properties that mainly derives from brown and white adipose tissue in humans (59). Yamamoto et al. demonstrated that resistin production in OSA patients can be enhanced by hypoxic stress during sleep and could mediate inflammatory processes (60). An experimental study showed that the mRNA levels of resistin were significantly increased in response to intermittent hypoxia (61). In addition, CIMT is positively correlated with serum resistin levels (56). However, a null significant difference in the serum resistin levels between the OSA group and controls has been demonstrated, and the serum resistin level is not associated with IMT (62).

To our knowledge, this is the first study to apply ultrasound RF data techniques to assess local arterial remodeling among OSA patients. We also investigated the relationship between adipokines (chemerin, adiponectin, SFRP5, and apelin) and arterial stiffness among OSA patients with standard polysomnographic data.

However, the study has some limitations. First, the sample size was small. Therefore, the relationship between the adipocytokines and the severity of OSA could not be established. Second, as the insulin levels were not detected, the relationship between insulin resistance and IMT/arterial stiffness could not be assessed. Third, the waist/hip ratio was not measured and the only BMI criterion may inaccurately estimate the degree of overweight.

In conclusion, we found that the quantitative IMT and carotid arterial elasticity of OSA patients were significantly worse and that SFRP5 levels decreased significantly in OSA patients. Adiponectin correlated with arterial stiffness among OSA patients but the relationship disappeared after a multivariable adjustment. Furthermore, age is the main independent factor correlated with quantitative IMT and arterial stiffness and that AHI and mini SaO_2_ were also independently associated with arterial stiffness. These results suggest that it is important to treat young OSA patients in order to reduce the risk for arterial stiffness, however, the role of adipocytokines requires further investigation.

## Data Availability Statement

The clinical data used to support the findings of this study are available from the corresponding authors upon request.

## Ethics Statement

The studies involving human participants were reviewed and approved by the Internal Review Board of the Institutional Ethics Committee of the Shanghai Jiao Tong University Affiliated Sixth Hospital. The patients/participants provided their written informed consent to participate in this study.

## Author Contributions

FS and HY designed the study. JZ, ZS, and JC performed the collection and handling of the data. HX, YQ, and HZ analyzed the data and wrote the manuscript. JG and SL revised the manuscript. All authors discussed the data and accepted the final draft.

### Conflict of Interest

The authors declare that the research was conducted in the absence of any commercial or financial relationships that could be construed as a potential conflict of interest.
